# Molecular and Functional Characterization of Three Odorant-Binding Protein from *Periplaneta americana*

**DOI:** 10.1371/journal.pone.0170072

**Published:** 2017-01-12

**Authors:** Zhao-Qun Li, Peng He, Ya-Nan Zhang, Shuang-Lin Dong

**Affiliations:** 1 Key Laboratory of Tea Biology and Resource Utilization, Ministry of Agriculture, Tea Research Institute, Chinese Academy of Agricultural Science, Hangzhou, China; 2 State Key Laboratory of Green Pesticide and Agricultural Bioengineering, Ministry of Education, Guizhou University, Guiyang, People's Republic of China; 3 College of Life Sciences, Huaibei Normal University, Huaibei, China; 4 College of Plant Protection, Nanjing Agricultural University/Key Laboratory of Integrated Management of Crop Diseases and Pests (Nanjing Agricultural University), Ministry of Education, Nanjing, China; USDA Agricultural Research Service, UNITED STATES

## Abstract

The American cockroach, *Periplaneta americana*, is a vector of many pathogenic organisms associated with human diseases. Olfaction plays a crucial role in guiding cockroach behaviors and contributes to their ability to transmit pathogens. Odorant binding proteins (OBPs), abundant in the insect olfactory sensilla, are important for insect olfaction. In this study, three *OBP* genes, *PameOBP1*, *2* and *3*, were cloned from *P*. *americana*. Sequence alignment and phylogenetic analysis revealed that PameOBP1, 2 and 3 belong to the Minus-C OBP, Classic OBP, and Plus-C OBP subfamilies, respectively. Expression pattern and ligand-binding analysis showed that *PameOBP1* and *2* were specifically expressed in antennae, and exhibited high binding affinities (Ki < 2 μM) to farnesene, farnesol, 2-tridecanone, and tetradecane, suggesting roles in volatile perception. Conversely, *PameOBP3* was ubiquitously expressed in most of the tissues examined at high levels and displayed very weak binding affinities (Ki > 40 μM) for all 87 ligands tested. Our study provides insights into the functional diversity of *PameOBP* genes and provides some volatiles that can potentially be used in behavioral interference of *P*. *americana*.

## Introduction

A sophisticated and sensitive olfactory system is crucial for many important insect behaviors, such as feeding, mating, and oviposition. The periphery process of insect olfaction involves interactions between odorants and three major protein classes including soluble binding proteins [1], membrane bound receptors [2], and odorant degrading enzymes [3].

As a key component in insect olfaction, odorant binding proteins (OBPs) are abundant, small, water-soluble proteins found in the sensillar lymph surrounding the sensory dendrite, and are thought to bind and transport odorant molecules through the sensillar lymph to activate odorant receptors [2, 4, 5]. After nearly three decades of research, a large number of OBP sequences have been identified from insects of different taxa [1, 6]. OBPs have been further grouped into four subclasses according to the number of conserved cysteines. Most have six conserved cysteines that form three disulfide bonds, designated as “Classic OBPs”. Three other subclasses of OBPs (non-classical OBPs) are “Minus-C OBPs” with four conserved cysteines, “Plus-C OBPs” with eight conserved cysteines, and “Atypical OBPs” with more than eight conserved cysteines [7]. When compared with the many reports on Classic OBPs, studies of non-classical OBPs are rare.

*Periplaneta americana* is an abundant and obnoxious pest that is well adapted to various man-made structures, such as dwellings, hospitals, hotels and restaurants, and prefers sweet food, including fruit, beer, putrid sake, bread, and peanuts [8]. This insect acts as a vector for many pathogenic organisms that are related to a number of human diseases [9]. Considering their habitat and proximity to human beings, an olfaction-based behavioral interference strategy is preferable for sustainable control of the American cockroach. Studies on the major genes involved in olfaction will be helpful for both the elucidation of the molecular mechanisms underlying olfaction and development of new control strategies for *P*. *americana*. To date, no studies have reported the functional characterization of *P*. *americana* OBPs.

In the present study, three *OBP* genes from each of the three OBP subclasses were cloned from *P*. *americana*. Further expression pattern analysis and ligand-binding assays showed that PameOBP1 and 2 might be involved in olfactory reception, while PameOBP3 may have other functions. These results provide a molecular basis for a deeper understanding of olfaction and suggest several volatiles that can potentially be used as attractants or deterrents for *P*. *americana*.

## Methods

### Insects rearing and tissue collection

The *P*. *americana* used in this experiment were provided by professor Zhao-Jiu Han (the Military Medical Institute of Nanjing Command). Experimental insects were reared at 25 ± 1°C and 65 ± 5% relative humidity in the laboratory on flour and milk powder, water was provided. The virgin females and males, respectively, were used to collect antennae, wings, legs, cercus, midguts, mouthparts, heads, thoraxes and abdomens. Tissue samples were immediately frozen in liquid nitrogen and kept at −80°C until RNA isolation.

### RNA isolation and cDNA synthesis

Total RNA was extracted using SV Total Isolation System (Promega, Madison, WI, USA) according to the manufacturer’s instructions. RNA quality was checked by a spectrophotometer (NanoDrop 2000c, Thermo Fisher Scientific, USA). cDNA templates for quantitative real-time PCR (qPCR) were synthesized using Reverse Transcription System (Promega, Medison, WI, USA) following the user manual, and cDNA template for RACE was synthesized using the SMARTer^™^ RACE cDNA Amplification Kit (Clontech, Mountain View, CA).

### cDNA cloning, sequence alignment and phylogenetic analysis

To obtain the complete sequence of *PameOBPs*, the partial sequences identified by our previous study [10] were used to design gene-specific primers for 5’- or 3’-end RACE. The specific primers used in RACE and RT-PCR were listed in S1 Table.

An amino acid sequence alignment of the three PameOBPs with OBPs from other insects was created using CLUSTALX 2.0 [11] and visualized by Jalview 2.4.0 b2 [12]. The three PameOBPs along with OBPs from *Blattella germanica*, *Rhyparobia maderae*, *Holotrichia parallela*, *Zootermopsis nevadensis*, Coptotermes formosanus, *Oedaleus asiaticus*, *Locusta migratoria manilensis* were used to construct a phylogenetic tree based on the amino sequences. Phylogenetic tree was constructed using the neighbor-joining in MEGA6 at default settings and 1000 bootstrap replicates [13].

### qPCR and data analysis

The qPCR was performed in a Mastercycler ep realplex (Eppendorf, Hamburg, Germany) with gene specific primers (S1 Table) designed based on *PameOBP* nucleotide sequences using Beacon Designer 7.7. *P*. *americana actin mRNA* (AAM77467) and *ADP-ribosylation factor* were used as reference genes [14, 15]. The expression levels of the tested mRNAs were measured using GoTaq qPCR Master Mix (Promega, Madison, WI, USA) according to the minimum information for publication of qPCR Experiments [16]. The relative expression level of the mRNAs for *PameOBP* genes were calculated according to the 2^−ΔΔCq^ method [17]. Each reaction was run in three technical replicates (every technical replicate had same reaction system) with three independent biological replicates. Every biological replicate of tissue samples was from 15 female and 15 male *P*. *americana*. Additionally, templates diluted into five-fold series were used to construct a relative standard curve to determine the PCR efficiencies.

### Expression and purification of the recombinant protein

The PameOBPs were expressed by using *Escherichia coli* expression system. To generate properly folded protein, the signal peptide predicted by SignalIP 4.1 [18] was removed. The two PameOBPs were amplified by gene special primers (S1 Table) with the cDNA as the template. The purified PCR products were ligated into expression vector pGEX-4T-1 using In-Fusion^®^ HD Cloning Kit (Clontech, Mountain View, CA) according to the manufacturer’s instructions. The recombinant plasmid was transformed into *E*. *coli* BL21 (DE3) cells, and then expression and purification was performed according to a previously reported protocol [19]. Recombinant proteins in the supernatant were purified by an affinity chromatography column GSTrap FF (GE Healthcare, Piscataway, NJ, USA). The GST tags were cleaved from recombinant proteins using 400 μL of thrombin (1U/μL) while loaded on GSTrap FF column at 25°C for 10 h and allowed 12 h. After that, the recombinant proteins were eluted with PBS buffer. Finally, the eluted proteins were desalted using HiTrap Desalting (GE Healthcare), lyophilized, and stored at −80°C until use.

### Competitive fluorescence binding assay

Emission fluorescence spectra were measured on a Hitachi F-4500 fluorescence spectrophotometer following our previous studies [20]. Firstly, dissociation constants of the PameOBPs for N-phenyl-1-naphtylamine (1-NPN) were calculated to determine the suitability of 1-NPN as a fluorescence reporter. After the determination of the reporter, the affinities of the PameOBPs for each ligand were measured using 2 μM 1-NPN as the fluorescent reporter and 0.25–2.5 μM or 2–40 μM as competitor, in at least three technical replicates. The binding data were analyzed following our previous studies [21].

## Results

### Identification and characterization of three *PameOBPs*

**Fig 1:**
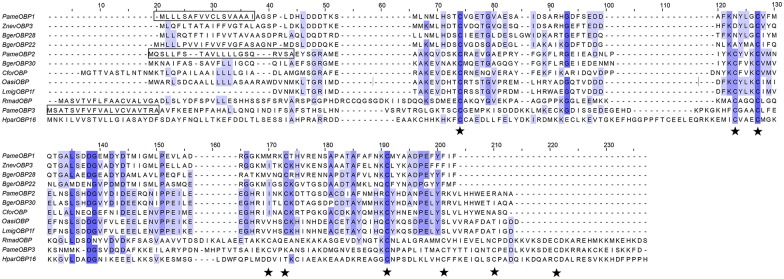
Alignment of PameOBPs amino acid sequences. Predicted signal peptides are boxed, and conserved cysteines are labeled with red pentagrams.

**Fig 2:**
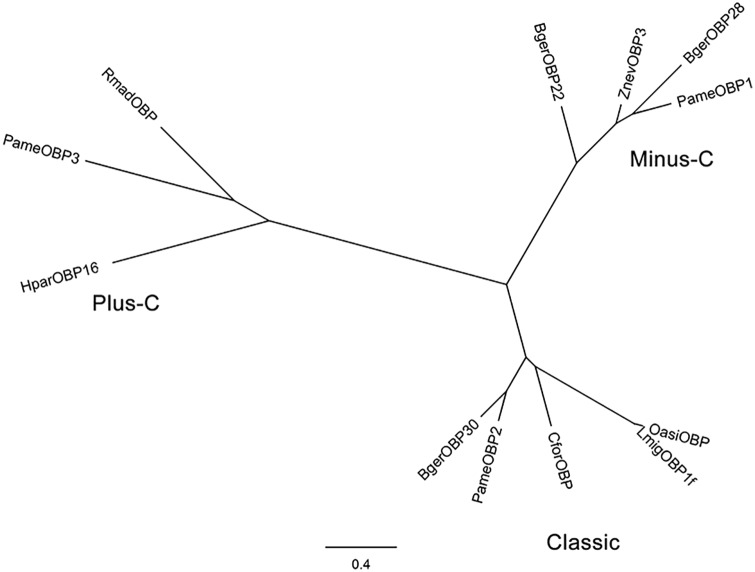
Phylogenetic tree of PameOBPs and OBPs from other species. Values at nodes indicate the bootstrap percentages based on 1,000 replicates, and branches with bootstrap values above 50% are marked. PameOBPs are highlighted in red.

Full-length cDNA sequences of the three *PameOBPs* (*PameOBP1*, *2* and *3*) were obtained by RACE and deposited in GenBank with the accession numbers ACI30685, ACI30686, and ACI30687, respectively. The mature *PameOBP1*, *2* and *3* encoded respectively 139, 149, and 215 amino acids with a predictable signal peptide. Sequence alignment and phylogenetic analysis revealed that these three PameOBPs belong to different OBP subfamilies. PameOBP1 is a member of Minus-C OBP as it contains only four conserved cysteine residues; PameOBP2 is a Classic OBP, with the typical six-cysteine motif; and with 8 conserved cysteine residues, PameOBP3 belongs to the Plus-C OBP subfamily (Figs 1 and 2).

### Tissue expression profiles of *PameOBP* genes

**Fig 3:**
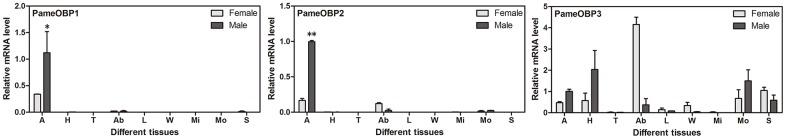
Relative mRNA expression levels of the five *PameOBPs* in different adult tissues. Error bars represent standard errors. A: antennae, H: heads, T: thoraxes, Ab: abdomens, L: legs, W: wings, Mi: midgut, Mo: mouthparts, S: cercus. *: 0.01<P<0.05, **: P<0.01,Student's t-test.

qPCR was used to investigate the expression levels of the *PameOBPs* in different adult tissues of *P*. *americana* (Fig 3). Results showed that *PameOBP1* and *2* had similar expression profiles, while *PameOBP3* showed a distinct pattern. *PameOBP1* and *2* had dominant expression only in antennae and were more highly expressed in male antennae than female. *PameOBP3* was expressed in multiple tissues, with higher expression in female abdomens than in male abdomens and other tissues.

### In vitro expression of PameOBPs

**Fig 4:**
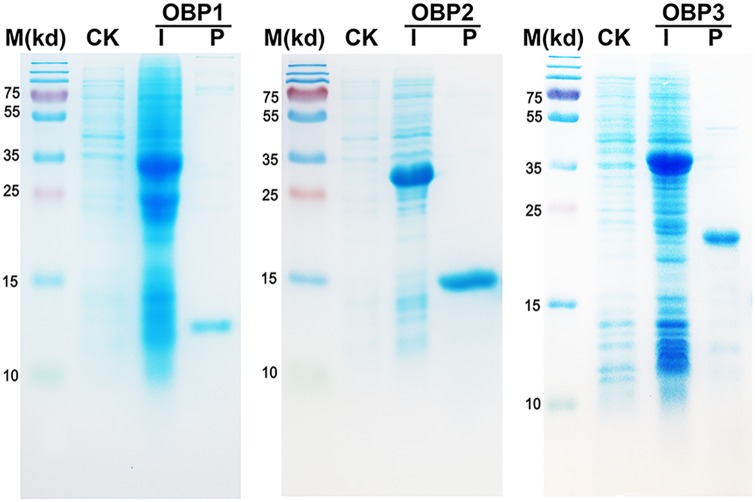
SDS-PAGE analyses showing the expression and purification of the recombinant PameOBPs. M: molecular markers; CK and I: bacterial cells before and after induction by IPTG, respectively; P: purified protein after cleavage by thrombin.

The three PameOBPs were successfully expressed as soluble proteins using a bacterial system. A GST-tag affinity column was used to purify the recombinant proteins, followed by treatment with thrombin to remove the GST-tag. About 15 mg of purified protein for each OBP was obtained from 1.0 L culture. The identity and integrity of the protein samples were confirmed by SDS-PAGE (Fig 4).

### Ligand-binding assays of the PameOBPs

**Fig 5:**
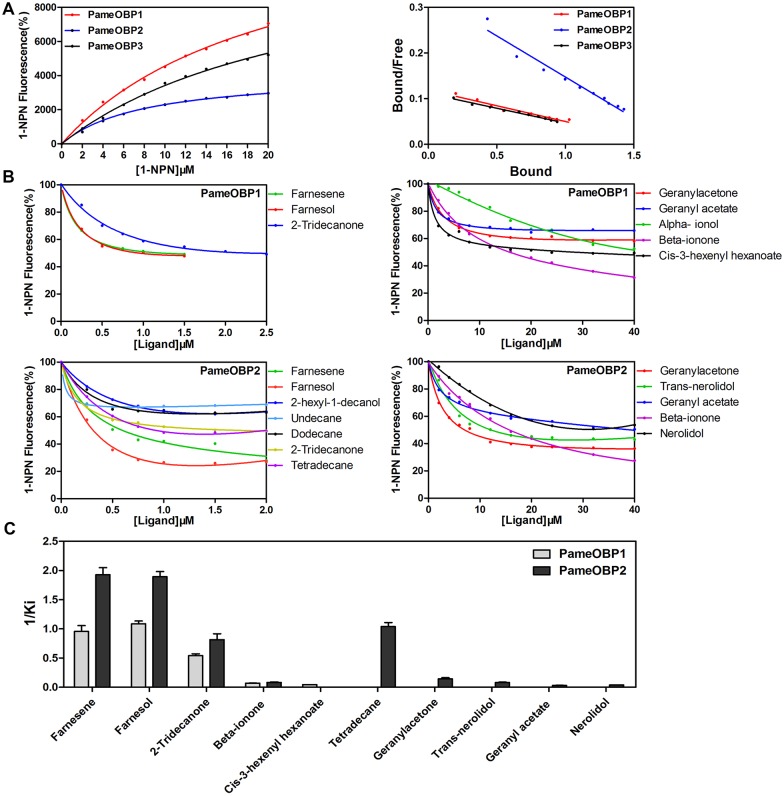
Ligand-binding assays of the PameOBPs. (A) Binding curves and Scatchard plots of PameOBPs for 1-NPN. (B) Binding curves of selected ligands. (C) Comparisons of binding affinities (indicated by 1/Ki) of PameOBPs with tested volatiles.

To investigate the role of three PameOBPs in the detection of odorants, we firstly tested the binding affinity of PameOBPs to fluorescent probe 1-NPN. Results showed that the three PameOBPs had different affinities for 1-NPN, with a dissociation constant of 20.54 μM for PameOBP1, 8.04 μM for PameOBP2, and 24.93 μM for PameOBP3. Saturation and linear Scatchard plots (Fig 5A) indicated single binding sites in the proteins with no allosteric effects. We were, therefore, able to use the fluorescence competitive binding assay to determine the binding affinities of these three PameOBPs to 87 odorants from apple, grape, peach, pear, the juice of these fruits, and beer [22–25].

The binding affinities of the three PameOBPs to compounds with binding affinities (Ki<40 μM) were calculated based on the binding curves (Fig 5B and 5C) and listed in Table 1. Different binding spectra were observed for the three proteins. Four ligands showed relatively higher binding affinities (Ki < 20 μM) for PameOBP1 and 2, while three additional ligands bound more strongly to PameOBP2. In particular, farnesene, farnesol, and 2-tridecanone showed very high binding affinities (Ki < 2 μM) for both PameOBP1 and 2, while tetradecane also bound strongly to PameOBP2 (Ki = 0.96 μM) (Table 1). PameOBP3 displayed only weak binding (Ki > 40 μM) with all 87 ligands tested.

**Table 1:** Binding data of the recombinant ParmOBP1 and PameOBP2 with different odorants.

Ligand	PameOBP1		PameOBP2	
	IC_50_ (μM)	Ki (μM)	IC_50_ (μM)	Ki (μM)
Farnesene	1.14±0.12	1.04±0.10	0.64±0.08	0.52±0.03
Farnesol	1.01±0.09	0.92±0.06	0.65±0.07	0.53±0.06
2-Tridecanone	2.02±0.16	1.85±0.17	1.52±0.16	1.23±0.08
Beta- ionone	15.77±2.1	14.44±1.6	15.05±1.1	12.17±0.9
Cis-3-hexenyl hexanoate	24.62±1.9	22.54±2.6	>40	>40
Tetradecane	>40	>40	1.19±0.09	0.96±0.10
Geranylacetone	>40	>40	8.62±0.68	6.97±0.72
Trans-nerolidol	>40	>40	15.55±1.30	12.57±0.98
Geranyl acetate	>40	>40	39.97±3.16	32.57±2.95
Nerolidol	>40	>40	32.90±4.21	26.61±2.79

Notes: IC_50_ values of <40 μM were obtained based on the binding curves. Other tested compounds (not listed) that have high Ki values (>40 μM) for all of the OBPs are beta-caryophyllene, alpha-phellandrene, (+/-)-alpha-pinene, 2-pentadecanone, (+)-beta-pinene, ocimene, (-)trans-caryophyllene, ethylbenzene, indole, naphthalene, cumene, tridecane, undecane, dodecane, octadecane, heptadecane, tridecane, benzyl alcohol, 1-hexanol, 2-hexyl-1-decanol, cis-3-hexen-1-ol, cis-2-hexen-1-ol, geraniol, (+/-)-linalool, eucalyptol, alpha-ionol, 2-heptanol, (+)-cedrol, linalool, trans-3-hexen-1, methyl anthranilate-ol, butyl formate, caproyl acetate, pentyl acetate, ethyl propionate, ethyl benzoate, octyl acetate, octyl aldehyde, cis-3-hexenyl acetate, trans-2-hexenyl acetate, phenylacetaldehyde, beta-cyclocitral, (+)-carvone, damascenone, 6-methyl-5-hepten-2-one, 2-heptanone, (±)-camphor, acetophenone, fodecyl aldehyde, decanal, hexyl butyrate, (+)-limonene oxide, (E3)-hexen-1-ol, camphene, (R)-(+)-limonene, phenethyl alcohol, 3-hexanol, Cis-3-hexenol, benzyl acetate, phenethyl acetate, cis-3-hexenyl acetate, ethyl butyrate, trans-2-hexenyl butyrate, phlorizin dehydrate, heptyl acetate, methyl salicylate, butyl acetate, nonyl acetate, isoamyl acetate, cis-3-hexenyl butyrate, benzaldehyde, hexanal, trans-2-hexenal, cis-3-hexenal, 3-hexanone, 2-hexanone, and undecanal.

## Discussion

This study is the first report on the expression patterns and ligand-binding properties of OBPs in *P*. *americana*. We cloned and characterized three *OBP* genes, *PameOBP1*, *2* and *3*, from *P*. *americana*. Based on the number of conserved cysteines, these three PameOBPs belong to different OBP subfamilies. PameOBP2 belongs to the Classic subfamily of OBPs that have six conserved cysteines. PameOBP1 is a member of the Minus-C OBP subfamily, members of which have lost two conserved cysteines, C_2_ and C_5_ compared with the Classic OBPs. PameOBP3 is a member of the Plus-C subfamily of OBPs that have two additional conserved cysteines [7]. This was also supported by phylogenetic analysis that showed PameOBP1, 2 and 3 grouping in the Minus-C OBP, Classic OBP, and Plus-C OBP clades, respectively, together with OBPs from other insects. This suggests that rapid evolutionary divergence of the three PameOBPs has occurred and may indicate different roles in odorant recognition [8].

OBPs that are mainly expressed in the major olfactory organs might be involved in olfactory functions [2, 26]. Two important classes of OBPs in Lepidoptera, pheromone binding protein and general odorant binding protein, are primarily expressed in antennae of both sexes and are involved in the detection of sex pheromone components and plant volatiles, respectively, in species such as *Helicoverpa armigera* [27], *Plutella xylostella* [28] and *Spodoptera exigua* [29]. Our qPCR data showed that *PameOBP1* and *2* were specifically expressed at high levels in antennae, and showed a strong male bias, suggesting that they might be involved in odorant or pheromone discrimination in *P*. *americana*. Conversely, *PameOBP3* was expressed non-specifically in different tissues, such as the head, antenna, mouthparts, and abdomen, indicating that it plays other, or multiple, physiological roles.

To confirm the functions suggested by the tissue expression profiles, the binding affinities of the three PameOBPs to volatiles from apple, grape, peach, pear, the juice of these fruits, and beer were determined using a fluorescent binding assay [22–25]. PameOBP1 and 2 showed high binding affinities (Ki < 20 μM) to four and seven volatiles, respectively, while PameOBP3 displayed weak binding (Ki > 40 μM) with all ligands tested.

Three volatiles, farnesene, farnesol and 2-tridecanone from apple, pear, grape and beer [24, 30, 31], had high binding affinities for both PameOBP1 and 2 with Ki < 2 μM. Beta-ionone is a fragrant compound found in the flowers and fruit of many plants [32, 33] and, therefore, plays an important role in host finding for many insects. Consistent with this, both PameOBP1 and 2 had high binding affinities for beta-ionone, with Ki values of 14.44 and 12.17 μM, respectively. High binding affinity for beta-ionone was also demonstrated in OBPs of many other insect species, such as *H*. *armigera*, *Sogatella furcifera*, and *Microplitis mediator* [34–36]. PameOBP2 also had high binding affinities for tetradecane and geranylacetone, two cashew apple juice volatiles [37], with Ki values of 0.96 and 6.97 μM, respectively. Conversely, PameOBP3 did not show a high binding affinity (Ki > 40 μM) for any of the tested compounds, suggesting that it functions in areas other than chemosensation, consistent with its expression in different tissues. The *in vivo* functions of these PameOBPs will be further confirmed through the generation and testing of RNAi insect lines.

## Conclusion

We cloned three *OBP* genes of different OBP subfamilies (Minus-C, Classic, and Plus-C OBP) from *P*. *americana*. qPCR and ligand binding assays suggest that *PameOBP1* and *2* function in olfaction, with PameOBP2 showing a broader odorant spectrum than PameOBP1, while PameOBP3 may function in areas other than olfaction. These results provide insight into the mechanism of olfactory recognition of *P*. *americana* and contribute to the development of insect attractants or deterrents that can be used in *P*. *americana* management.

## Supporting Information

S1 Table Primers used in RACE, qRT-PCR, and Vector construction. (DOCX)
