# Supplementary material for: Molecular and Functional Characterization of Three Odorant-Binding Protein from Periplaneta americana
Source: PLoS One. 2017 Jan 12;12(1):e0170072. doi: 10.1371/journal.pone.0170072 (PMC5232348; doi:10.1371/journal.pone.0170072)
Supplement: S1 Table — (DOCX) [file pone.0170072.s001.docx]

**S1 Table. Primers used in RACE, qRT-PCR, and Vector construction**

| Purpose | Primer name | Sequence (5’→3’) |
| --- | --- | --- |
| RACE PCR | PameOBP1-5’ | AGTACCTCTGGCAGCATCCCGATCAT |
|  | PameOBP1-3’ | GTGGCGGAGTCTGCCATTGACAGT |
|  | PameOBP2-5’ | GTCCGAACCAGTCGTGTCTTTGCACT |
|  | PameOBP2-3’ | TGAGCTGAACTCGCTCTCCCATGATG |
|  | PameOBP3-5’ | AACGCATCTTGGTCGACGGATCCAT |
|  | PameOBP3-3’ | CTTTTGCGCACGCTCTGCTTCAGAAT |
| qRT-PCR | Actin-F | TCCTGAAGAGCATCCTGTTCT |
|  | Actin-R | GAATCCAGCACAATACCAGTAGTC |
|  | ARF-F | TTGATGGTTGGTCTGGAT |
|  | ARF-R | CCTGAGTATTCTGAAAGTAGTG |
|  | PameOBP1-F | ACGATGGAGAAATGGACTACGA |
|  | PameOBP1-R | GTTGAAGGCGAAGGCAGTT |
|  | PameOBP2-F | CGTCAAGTGCGTGATGAATGA |
|  | PameOBP2-R | TCCGAACCAGTCGTGTCTT |
|  | PameOBP3-F | AATAAGCGACGACGACAAGAAG |
|  | PameOBP3-R | CAGGCAGCACCACAGAAG |
| Vector construction | PameOBP1-F | GGTTCCGCGTGGATCCGGTAGCCCGCTGGACCAT |
|  | PameOBP1-R | AAATTCCCGGGGATCCTCAGAAGATGAAGTAGAACTCGGG |
|  | PameOBP2-F | GGTTCCGCGTGGATCCCGAATATTCGGGCCGAGC |
|  | PameOBP2-R | AAATTCCCGGGGATCCTTAGGCGTTCGCACGTTCTT |
|  | PameOBP3-F | GGTTCCGCGTGGATCCGCAGTATTCAAAGAGGAAAATCCTT |
|  | PameOBP3-R | AAATTCCCGGGGATCCTCAGTCGAACTTCTTGCTAATTTCC |
